# The Hip Joint

**Published:** 1877-04

**Authors:** E. R. Barnes


ART. II.—*The Hip Joint.* By E. R. BARNES, M. D. Read before the Buffalo Medical Association.

[I must apologise for presenting a paper which is purely elementary in character. I have not attempted to advance anything new or original but only what is generally accepted by authorities. I have not attempted to instruct, but only to review, and while this may be tedious to those who have given much attention to surgical subjects, yet to the general practitioner, who is liable to be summoned in cases of hip injury, such a review may not be objectionable. I have reason to believe this is true, and for this reason, together with the interest recently excited on this subject and the variety of opinions expressed thereon, I venture to begin at the bottom, even if I do not get very near the top.]

The bones and parts of bones concerned in the formation of the hip joint, or of immediate interest in connection with lesions of the same are broadly, the os-innominatum and the os-femoris. The parts thereof of interest as aforesaid are—of the os-innominatum—the acetabulum, the dorsum illii above, the greater and lesser sacro-sciatic notches posteriorly, the body of the pubic bone in front, and below the obturator foramen, bounded as it is by the ischium, and its ramus and the pubic bone and its ramus.

The acetabulum is a hemispherical depression, formed at their points of junction, from portions of the three bones into which originally the os-innominatum was divided. It is surrounded by an irregular rim, strongest above, which is about two inches in diameter, and which makes the greatest depth of the contained cavity about three-fourths of an inch. This rim is continuous, except where it is interrupted by the cotyloid notch, which opens downwards, and a little forwards towards the thyroid foramen. The inclined position of the acetabulum insures firm support to a body entering it in the direction of the os-femoris.

The acetabulum is lined with an articular cartilage except at the attachment of the ligamentum teres, which cartilage extends beyond the rim of the acetabulum about one-quarter of an inch or more, increasing the depth of the cavity by that amount. This extension of the acetabular cartilage is called the cotyloid ligament, because it is rendered very strong by containing within its substance an interlacing network of strong compact fibrous tissue, which is firmly attached to the bony rim. It is inclined inwards, so that at its narrow edge the acetabulum is narrowed, and embraces the cartilaginous head of the femur. It surrounds the acetabulum, except where it becomes continuous with the strong transverse ligament which bridges the cotyloid notch, and completes the continuity and symmetry of the acetabular cavity. It is, like the acetabular rim, much thicker and stronger above than elsewhere.

The parts of the os femoris which are of interest in this connection are the head, the neck, and the upper part of the shaft, including the greater and lesser trochanters, connected by the greater and lesser intertrochanteric lines. These parts consist of cancellated tissue, covered by a layer of compact tissue, of greater or less thickness, according to situation. The head, which is somewhat more than a hemisphere, is covered by articular cartilage, and is supported by the neck, which passes obliquely downwards, until it abuts against and is incorporated into the shaft. The base of the neck is determined or bounded by the intertrochanteric lines, and by the trochanters major and minor, and is very much thicker from above downwards, though not from before backwards, than at the attachment of the neck to the head. The under-surface of the neck approaches the shaft in the form of an arch. The compact tissue forming this under-surface is everywhere, for obvious reasons, very much thicker than that of the upper surface. It is thinnest where it is attached to the head, and grows progressive thicker until it is incorporated into the compact tissue of the shaft. The anterior and posterior compact tissue coverings of the neck are of about equal thickness above, but as we approach the lower surface, where the anterior wall is much the thicker of the two, the posterior wall becomes thinner, until it is reduced almost to the thinness of paper at its insertion into the posterior intertrochantric line.

As to the internal construction of these parts, it is only necessary to call attention to the arrangement of the cancelli, so as best to receive the weight, and transmit it by means of direct lines, or pillars, or braces and stringers, to the support of arches to have a fine instance of the mechanism of nature. Dr. Bigelow has pointed out an interesting fact as to this cancellous structure. It is that the posterior inferior wall of the neck is prolonged into the cancellous structure, beneath the posterior intertrochanteric ridge, constituting what he calls the true neck, while the external paper-like shell, curving outwards to join the ridge, cannot be considered the main structure. To the fact of this conformation he attributes much importance in connection with some of the phenomena of impacted fracture of the neck.

The ligaments of interest are the greater and lesser sacro-sciatic ligaments, forming the foramina of the corresponding names; the ligamentum teres, whose function it is to limit rotation outwards, and to check adduction when the limb is flexed, but which is sometimes rudimentary or even absent altogether; the illio femoral ligament, passing fan-shaped from the anterior inferior spine of the illium to the anterior intertrochanteric line, divided as it is essentially into two powerful fasciculi which are inserted into the opposite extremities of the anterior line, whose agency, as shown by Bigelow, who calls it the Y ligament, is more important in controlling the phenomena of dislocation than that of any other ligament or of any muscle, and it might perhaps be said than that of all others combined; and lastly, the capsular ligament, originating from the margin of the acetabulum external to the cotyloid ligament, surrounding the neck at its base anteriorly, but varying in its insertion posteriorly, being generally said to be inserted here at the middle of the neck. It is much thicker above and in front than elsewhere. The inner layer of the capsular ligament, reflected, becomes the capsule, or rather the periosteum surrounding the neck within the joint.

If we now lay open an imaginary capsular ligament, we expose an extensive cavity lined with synovial membrane, which, commencing at the head of the femur, covers the neck as far as the insertion of the capsular ligament, on to the internal surface of which it is reflected; thence, passing over the external surface of the cotyloid ligament, it dips into the acetabulum, and, covering the ligamentum teres, reaches again the head of the bone whence it started, so to speak. This synovial membrane or sac does not extend as far outwards as would appear from the external insertion of the capsular ligament in front, owing to the great thickness of the latter anteriorly and above, and the difference in points of insertion between its superficial and deep layers. That portion of the neck only which is included in the synovial sac, is regarded as intra-capsular; hence a fracture of the neck may be wholly within the capsule, without being wholly intra-capsular in a surgical sense. This point is of importance in connection with the question of bony union after intra-capsular fracture.

As to the muscles it is only necessary to say that numerous and powerful muscles surround and are connected with the bony structure in this region, aiding to maintain their proper relations, controlling movements in a normal state, and influencing abnormal phenomena. To refer to these in detail would consume too much space, of which I have already taken too much under this head. I will only call attention to the obturator internus, which, passing out of the pelvis by the lesser sacro-sciatic notch, is inserted into the upper portion of the great trochanter posteriorly. Its structure is such as to render it very strong, and Bigelow has shown that it has important relations to fracture of the neck, but especially to dislocation.

Passing now to the subject of fracture of the neck of the femur, I would state that the head, covered with its cartilage, fits so exactly into the cotyloid cavity that atmospheric pressure is stated by Holmes to be one of the agencies in retaining it in place. There is no room for yielding. The natural mode of providing against shock to the joint structures, is by the transmission of force through angles, as that between the foot and leg at the ankle, between the leg and thigh at the knee, and between the thigh and body at the hip joint. A fall upon the foot in the erect position, with muscles rigid, may readily injure the joint structures. So in general it may be said that it does not require a strain, or blow, or shock, of the greatest severity to fracture the neck of the femur, provided the force acts without the intervention of angles, with the elasticity afforded by muscular support. In accordance with this principle, fracture of the neck of the femur is generally caused by a fall upon the foot as stated, or upon the knee, or upon the region of the great trochanter.

The liability to this accident increases with age. Dupuytreen says he never saw a fracture of the neck of the femur in a child. Among the earliest cases recorded is one by Sabatier, in a child of fifteen years. Hamilton says this is the earliest case he has seen recorded. Before fifty years of age fracture of the neck is comparatively rare in a healthy subject, except as the result of a violent blow upon the great trochanter, as in the case of a person falling from a height. After fifty the liability increases in proportion to age, so that beyond sixty years progressively, a very slight cause will produce fracture of the neck. The ease with which this occurs, is due to the changes in nutrition, senile atrophy and sometimes fatty degeneration of the neck, which occur in advanced life, and not to the relative increase of the earthy material of the bone, as is sometimes stated, which Mr. Barnsby Cooper has shown, is on the contrary diminished in the neck, although increased in the shafts of the same specimens.

The relative frequency of extra and intra-capsular factures is not determined, but it may be said in general that before fifty the presumption is all in favor of an extra capsular fracture, and that afterwards progressively with age, more especially after sixty years, the presumption is in favor of an intra-capsular fraction; meaning here a fracture wholly within the capsule, or partly within and partly without the capsule. If a man over sixty years old receives a slight fall, the presumption is in favor of an intra-capsular fracture as against an extra capsular or a dislocation. The exception to this statement is, when a severe, direct blow is received on the outer side of the great trochanter.

M. Rodet, by experiments, claims that one may tell the the situation and direction of this fracture by a knowledge of the direction in which the force has acted. As his conclusions seem to be accepted as in the main correct by the highest authorities, they may be thus stated: When a person has fallen upon the foot or knee, the force, acting vertically, will produce an oblique intra-capsular fracture. When the blow is upon the front of the trochanter the force, acting from before backwards, produces a transverse intra-capsular fracture. When the back of the trochanter is struck the force, acting from behind forwards, will produce a fracture partly within and partly without the capsule ; while if the force act transversely, *i. e.*, fairly against the outer side of the trochanter, the fracture will be wholly extra capsular. It may be added that extra capsular fracture is generally produced by greater force than intra-capsular.

These points are of accessory value in the diagnosis. A distinction cannot always be made between all the different forms of fracture by the symptoms, and by manipulation, which must be guarded. Yet a diagnosis is important in enabling us to make a prognosis, for it may be said that an exra capsular fracture, under favorable circumstances, will generally unite by bone ; a fracture partly within and partly without the capsule may often unite by bone, while it has not been proven that bony union of a wholly intra-capsular fracture is anything but phenomenal, if it has been proven to take plaee at all. The difficulty of proving the existence of bony union in intra-capsular fracture, from the specimens purporting to be examples of such union, consists mainly in the impossibility of establishing the location of the original fracture, absorption having removed more or less of the fragments of the neck, and caused the insertion of the capsule to recede from its normal position, before union took place, thus failing to show anything but an extra capsular union of an intra-capsular fracture, as pointed out by Dr. George K. Smith, in his valuable monograph on the insertion of the capsular ligument of the hip joint, and its relations to bony union in intra-capsular fracture. A diagnosis will also aid in the treatment, as to time of continuance in bed, extension, &c.

Fractures of the neck vary greatly in degree. Partial fractures are recognized from specimens, as existing ; also, intra-capsular fractures of the neck, without laceration of the periosteum : and this is one of the conditions in which Sir Astley Cooper claims bony union to be possible. These conditions could not be recognized as such during life. But fractures of the neck are generally complete, and may occur at any part of the neck. The strong capsular ligament is not usually ruptured at first, or but slightly so. The periosteum of the neck is generally ruptured, and the fragments of the neck more or less displaced.

Intra-capsular fractures may be impacted, but I think this is rather the exception. Extra capsular fractions are said to be always impacted. In this latter class of fractures, the neck, usually the posterior portion of it, is driven into the cancellated tissue of the shaft, often splitting off one of the trochanters, or, with greater force, more extensively comminuting the bone. In the latter case impaction may be immediately liberated. Impaction may also be liberated in other ways.

The mode of union of these fractures is by ligament, or bone, or no union of any kind may occur. Ligamentous, or fibrous union, may be so close as to require maceration to distinguish it from bony union; or the ligaments may be long and allow a play of the fragments upon each other. Where bony union takes place there is generally more or less absorption of the neck. Where no union whatever takes place, absorption of the neck also occurs, and the weight of the body is sustained by the ligaments and muscles connected with the joint. Bigelow presents a specimen which had been a case of old ununited fracture of the neck, in a subject whose weight in walking had been sustained chiefly between the outer branch of the Y ligament in front, and the obturator internus behind. This, he states, is probably the usual condition of patients after this injury, where the shaft of the femur moves freely upon the detached head of the bone.

I will now refer to some of the symptoms attending fracture of the neck. First—Deformity. In a recent intra-capsular fracture there may be an increased prominence of the hips from the presence of effusion, or from the position of the bones, or this symptom may be wanting, or there may even be a slight depression on that side. In extra capsular fracture there is usually less prominence than on the uninjured side. When there is much impaction this is very apparent. Secondly—Rotation. In impacted fracture the trochanter rotates through an arc of a circle of which the head of the bone is the center. In detached fracture it rotates on the axis of the shaft. Thirdly—Crepitus. This is often not attainable, because the fragments are not in apposition and cannot be brought into a normal relation, or when in such relation, the serrated edges of the neck may cause the acetabular fragment to move with it, or because a portion of torn ligament may be interposed between the fragments, or because there is impaction, which it is important not to disturb. Fourthly—Loss of power over the limb. The patient, in a recumbent position, cannot lift the limb by a voluntary effort, although there is no resistance to any motion of the limb by the surgeon, except from the effort of the patient to avoid pain from motion. There is unrestricted mobility under chloroform. Nevertheless, if the capsular ligament remains entire, or if there is firm impaction. the patient may walk some distance after the injury, at least with assistance. There is often semi-flexion of the knee, in recent intra-capsular practice. Fifthly—Pain and tenderness at the seat of injury. Sixthly—Eversion and shortening. I mention these together because they are sometimes due to the same cause, sometimes not. Where there is no impaction, shortening is due to muscular contraction. This shortening may be at first greatly limited by the untorn reflexion of the capsular ligament, or by the latter itself, to a less degree, but the shortening subsequently becomes greater, by the yielding of these structures, or, if bony union ultimately takes place, by the absorption of the neck. Eversion takes place in unimpacted fracture simply because that is the natural position of the limb when left to itself. The agency of the external rotators in producing eversion is greatly lessened by the destruction of their centre of motion by the fracture of the neck of the former. Their action is more directly backwards than rotatory, as suggested by Erichsen.

In impacted fracture, eversion takes place in consequence of the posterior wall of the neck being the part impacted, and the shortening is caused by the depression of the head, which results from the peculiar mode of this impaction. Bearing in mind Bigelow's description of the posterior wall of the neck, referred to in the earlier part of this paper, I quote from him on this point: "In impacted fracture, the thin posterior wall is alone impacted, while the thick anterior wall, refusing to be driven in, yields only as a hinge, upon which the shaft rotates to allow the posterior impaction. . . . The hinge before alluded to is oblique, following the anterior intertrochanteric line. Were it vertical, by bending this hinge we would produce rotation without shortening. On the other hand if it were horizontal and transverse, bending it would produce shortening without rotation. But as it stands at an angle of 45°, the shaft rotating upon this broken interval is shortened in proportion to its rotation—or what is the same thing the neck is reflected upon its hinge downwards and backwards, till its axis, normally obliqne, may become even transverse, with great outward rotation of the shaft, and a shortening of perhaps two inches. This is probably the most common cause of shortening, though the head of the bone may be otherwise depressed." Slight impaction will produce slight eversion and shortening.

When there is no eversion, and even, rarely, inversion, the neck may be driven by great force directly into the cancellous tissue of the shaft with much splitting and comminution of the shaft, and the neck is variously depressed and shortened. By this injury the limb may be shortened three inches.

In recent unimpacted fracture the limb can readily be brought down to its normal length, which of course cannot be done in impacted fracture. Measurements are taken by a comparison of the distance between the anterior superior spinous processe of the illium and the mallcolus of the injured side with that on the opposite side to ascertain the existence and degree of shortening. Then, from the great trochanters to the inner condyles, or the lower borders of the patellæ; then from the patellæ to the inner malleoli, to ascertain that the shortening is neither in the thigh or leg. In marked shortening the great trochanter of the injured side is nearer the anterior superior spine of the illium than on the other side. I believe that careful measurements will show that it is also often nearer the median line posteriorly. Careful measurements of cases of old fracture of the neck with about two inches shortening, which I have recently made in the presence and with the aid of others, form the basis for this statement. This nearing both points could be caused by the shortening of the neck, and by the approximation of the lower angle of the triangle, which is formed between the three points indicated, to the two other angles, as is done by the moving upwards of the great trochanter. It can be shown also by triangles that the tilting of the pelvis does not perceptibly affect the relative measurements of the two limbs.

When a person with marked shortening from fracture of the neck is in a sitting posture, the projecting trochanter can be most readily felt by the hand, and it sometimes seems to be nearer the seat than the other.

In respect to treatment, perhaps the broad division made by Bigelow is the best, viz: into "impacted fractures of the base of the neck and unimpacted fracture of the rest of the neck, without regard to the capsule, a practical classification, embracing a majority of cases, and to which the other lesions may be regarded as exceptional." Bearing this in mind, and not failing to apply what knowledge we possess, as to the modes and probabilities of union, in the different conditions influencing this fracture, and we have our guide to treatment. Thus impacted extra capsular fracture requires immobility without extension. The remaining fractures in general require extension to approximate fragments, and immobility. Various appliances are used to attain these ends. In old and infirm persons too prolonged an attempt must not be made to procure union. In some cases even the patient must be relieved as soon as possible from confinement to bed without any attempt to secure union.

My paper has become so long that I have been unable to refer to any other lesions.

